# Interplay of Trapped Species and Absence of Electron Capture in Moiré Heterobilayers

**DOI:** 10.1021/acs.nanolett.3c01177

**Published:** 2023-06-23

**Authors:** Arnab Barman Ray, Arunabh Mukherjee, Liangyu Qiu, Renee Sailus, Sefaattin Tongay, Anthony Nickolas Vamivakas

**Affiliations:** † The Institute of Optics, University of Rochester, 480 Intercampus Dr, Rochester, New York 14627, United States; ‡ Arizona State University, 1151 S Forest Ave Tempe, Arizona 85281, United States; ¶ Center for coherence and quantum optics, Department of Physics, University of Rochester, 480 Intercampus Dr, Rochester, New York 14627, United States

**Keywords:** 2D materials, Moiré superlattice, interlayer excitons, optoelectronics

## Abstract

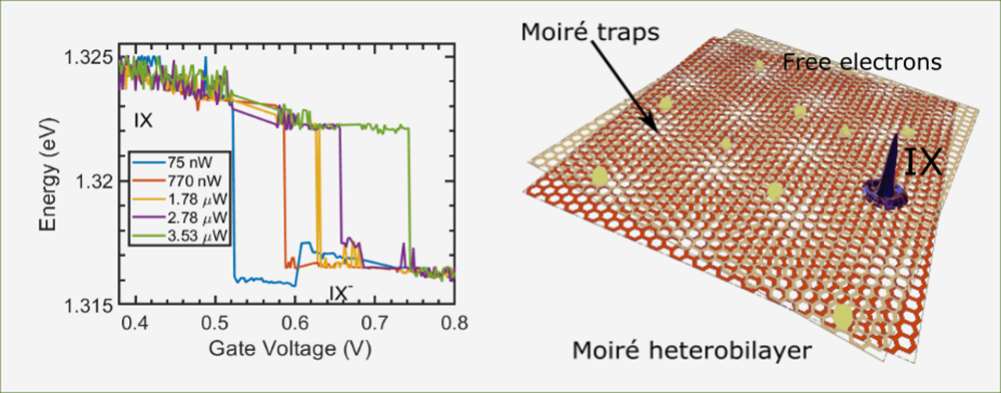

Moiré heterobilayers host interlayer excitons in a natural, periodic array of trapping potentials. Recent work has elucidated the structure of the trapped interlayer excitons and the nature of photoluminescence (PL) from trapped and itinerant charged complexes such as interlayer trions in these structures. In this paper, our results serve to add to the understanding of the nature of PL emission and explain its characteristic blueshift with increasing carrier density, along with demonstrating a significant difference between the interlayer exciton-trion conversion efficiency as compared to both localized and itinerant intralayer species in conventional monolayers. Our results show the absence of optical generation of trions in these materials, which we suggest arises from the highly localized, near subnanometer confinement of trapped species in these Moiré potentials.

Heterostructures, made by stacking monolayers of transition metal dichalcogenides^1−7^ (TMDCs), as well as other materials, such as insulating hexagonal boron nitride (hBN),^8,9^ have attracted significant scientific interest over the past decade. The possibilities of constructing different kinds of condensed matter systems^10−12^ through the layer degree of freedom and the choice of material have been a cornerstone of research in this direction. In recent years, it was found that bilayers, consisting of two monolayers of either the same (homobilayer) or different (heterobilayer) monolayers can host luminescent long-lived interlayer excitons.^13,14^ The longer lifetimes of interlayer excitons in these heterostructures, as opposed to intralayer excitons in monolayers, have made them a material of choice for excitonic applications.^15,16^ More recently, it has been found that two monolayers in contact with each other in a heterobilayer (hBL) develop a periodically varying potential that can trap these interlayer excitons.^17−20^ The structures, called Moiré superlattices, have a period that depends critically on the twist angle between these layers and the mismatch between the lattice period of the two layers themselves.^21,22^ These hBLs were shown to host single-photon quantum emitters at the Moiré trapping sites, which also offer a degree of “programmability” through the use of magnetic and electric fields, which can be used to tune the emission wavelengths^23^ and polarization.^24^

MoSe_2_/WSe_2_ hBLs are one of the most well studied TMDC Moiré systems.^25,26^ They are characterized by a large Moiré period (100 nm) with near 0° alignment, owing to the small lattice mismatch between the two monolayers. More recently, isolated trapped species have been observed. Trions, biexcitons, and evidence for higher order charged complexes have emerged.^27−29^ Beyond isolated emitters, at higher excitation intensities, different species of trions have been identified in this system.^30^ Moiré trions are very interesting as they allow a higher degree of nonlinearity in their interactions with light through a phase-space filling effect combined with Moiré localization effects.^31^ They may have potential for use in nonlinear photonic devices for quantum computing applications.^32,33^ In this regard, a thorough understanding of the physics of Moiré trions is desirable.

The photoluminescence (PL) emission from interlayer excitons in MoSe_2_/WSe_2_ Moiré heterobilayers has been studied in some detail, and three broad phases of excited state species were reported from diffusion-based studies—trapped excitons, itinerant excitons, and electron–hole plasmas at very high excitation intensities equivalent to carrier densities of the order of 10^13^ cm^–2^.^4,34^ However, at intermediate carrier densities in the range 10^10^–10^11^ cm^–2^, when the spectral lines from individual trapped emitters are no longer isolatable due to power broadening and higher densities of trapped emitters, the dynamics between different species are not clear. This state of the system, which we call the quantum ensemble regime,^30^ represents emission from a collection of different trapped species across the excitation spot.

Our results investigate this ensemble, and we observe, by normalizing the PL, that trions, excitons, and biexcitons exist in this regime and are seen as spectrally separated emission bands. We find a spectral weight transfer across the different species with increasing optical excitation density. The strong correlation of the trion PL with the amount of electrostatically doped electrons in the system indicates an absence of trion-exciton conversion at higher excitation fluxes typical of free and trapped intralayer excitons in monolayers.^35−37^ We note that the absence of optically generated trions is a feature that distinguishes Moiré systems from conventional monolayers that have been studied so far, with itinerant^35,38^ or defect-localized intralayer excitons.^36^ The blue shift observed in the PL at lower excitation intensities has been attributed to the dipolar repulsion between interlayer excitons across Moiré trapping sites.^30^ However, our results show that most of the blue-shift that has been observed consistently so far arises primarily from a spectral weight transfer from one species to the other with increasing excitation intensity.

We use a dry transfer technique to assemble a double hBN encapsulated MoSe_2_–WSe_2_ single-gated heterostructure with a PC stamp under a microscope. The hBN was obtained from 2D semiconductors. The bulk MoSe_2_ was n-type from HQ Graphene, and the WSe2 was prepared at ASU. The monolayers were aligned along precise straight edges, which allowed us to make luminescent hBLs, but without any control on whether the resulting stack would be R-type or H-type,^21^ although magnetic measurements in this sample confirm from the observed g-factors that the sample is R-type (Supporting Information). From the precision of our transfer setup and the luminosity of the interlayer PL signal, we estimate a small twist angle of 0–5° for our R-type samples. We note that for lattice-mismatched hBLs, the period of the Moiré superlattice is controlled mostly by the small lattice mismatch and depends less strongly on the exact twist angle. We used an n-doped monolayer to observe trions without any electrostatic doping. We assemble the hBL on top of a chip with 285 nm of thermally grown SiO_2_ on Si. The back gate consists of an FLG in contact with one of the 50 nm gold electrodes. The monolayers are also in contact with another electrode, allowing us to bias the device as a parallel plate capacitor. An accurate voltage source (ANC 300 controller) is used to bias the device. Figure 1(b) shows an optical micrograph of the device under consideration.

**Figure 1:**
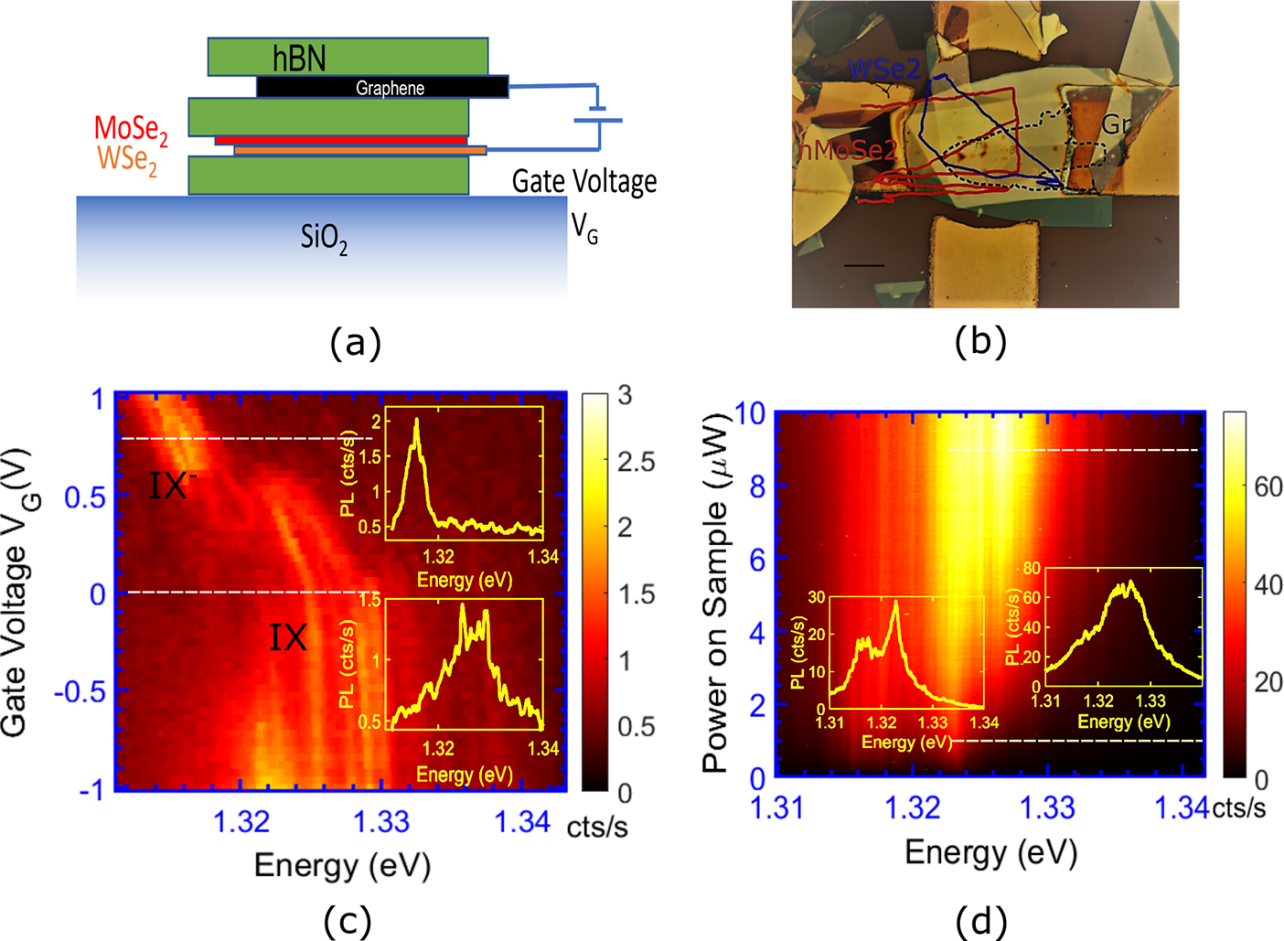
(a) A schematic of the kind of devices investigated, (b) optical image of the first sample, the scale bar is 10 μm, (c) PL from interlayer excitons and trions showing a transition with increased doping voltage at an excitation intensity of 20 nW. The insets are a plot of the PL spectra at *V*_G_ = 0.8 V(top), *V*_G_ = 0.0 V(bottom). (d) Evolution of the PL with increasing laser intensity, with insets at 1 μW (left) and 9 μW (right).

The measurements were carried out using a home-built confocal microscope. A PID stabilized 532 nm DPSS laser is focused into a submicrometer diameter spot using a 0.82 NA objective lens in a closed-cycle cryostat (AttoDry 1000) at 4 K equipped with a superconducting magnet. The PL emitted is collected by the same objective and coupled into a multimode fiber. The collected PL is analyzed by using a Princeton Instruments spectrometer (Acton SP-2750i) and an LN2 cooled Pylon CCD camera. We used an intensity calibrator (IntelliCal, Princeton Instruments) to account for the drop in the quantum efficiency of the Si CCD camera at near-IR wavelengths.

First, we investigate the PL from the sample at low carrier densities when the narrow emission from the individual emitters is well differentiated. The PL we investigate from this sample (R-type) arises from the singlet exciton.^40,41^Figure 1(c) shows a voltage sweep where the voltage is systematically incremented until we see a sharp transition from trapped interlayer excitons to trions. The narrow emission peaks are characteristics of these quantum emitters with individual line widths < 100 μeV. We then focus on how the PL evolves with increasing laser intensity, as shown in Figure 1(d). We note a blue-shift in the observed PL by about 8 meV. The line width of the emission is around 5–6 meV. We note the nonlinear evolution of the PL with excitation intensity. For the highest excitation intensities considered in this paper, we estimate a charge carrier density of 5 × 10^11^ cm^–2^, well within the regime where the emission is dominated by Moiré trapped excitonic species.^34^ We arrive at this estimate by using the steady state equation, *n* = *αIτ* and a carrier lifetime of τ = 2 ns,^13^ an absorption coefficient^42^ of α = 0.10 at 2.33 eV in a spot size of area 1 μm^2^.

We normalize the PL spectra in Figure 2(a) to gain further insight. The PL emission at a given excitation intensity is normalized by dividing the spectrum by the observed peak counts. We see that normalizing the PL resolves the emission into three distinct bands. The energy difference between the first two bands is obtained by subtracting the energy difference between the center of the two bands and is found to be around 5–6 meV. The energy difference between the second and third band is around 3–4 meV. We note that these values are consistent with reported values of the binding energy for trion,^29,30^ and the additional energy that the trapped biexciton exhibits due to intratrap dipolar repulsion, respectively.^27^ The binding energies for the trion are much lower than are seen for intralayer trions due to the effects of localization on the trion wave function. Similarly, the spatial proximity of two excitons in the same Moiré trap and the extreme localization causes the biexciton to emit at a higher energy.

**Figure 2:**
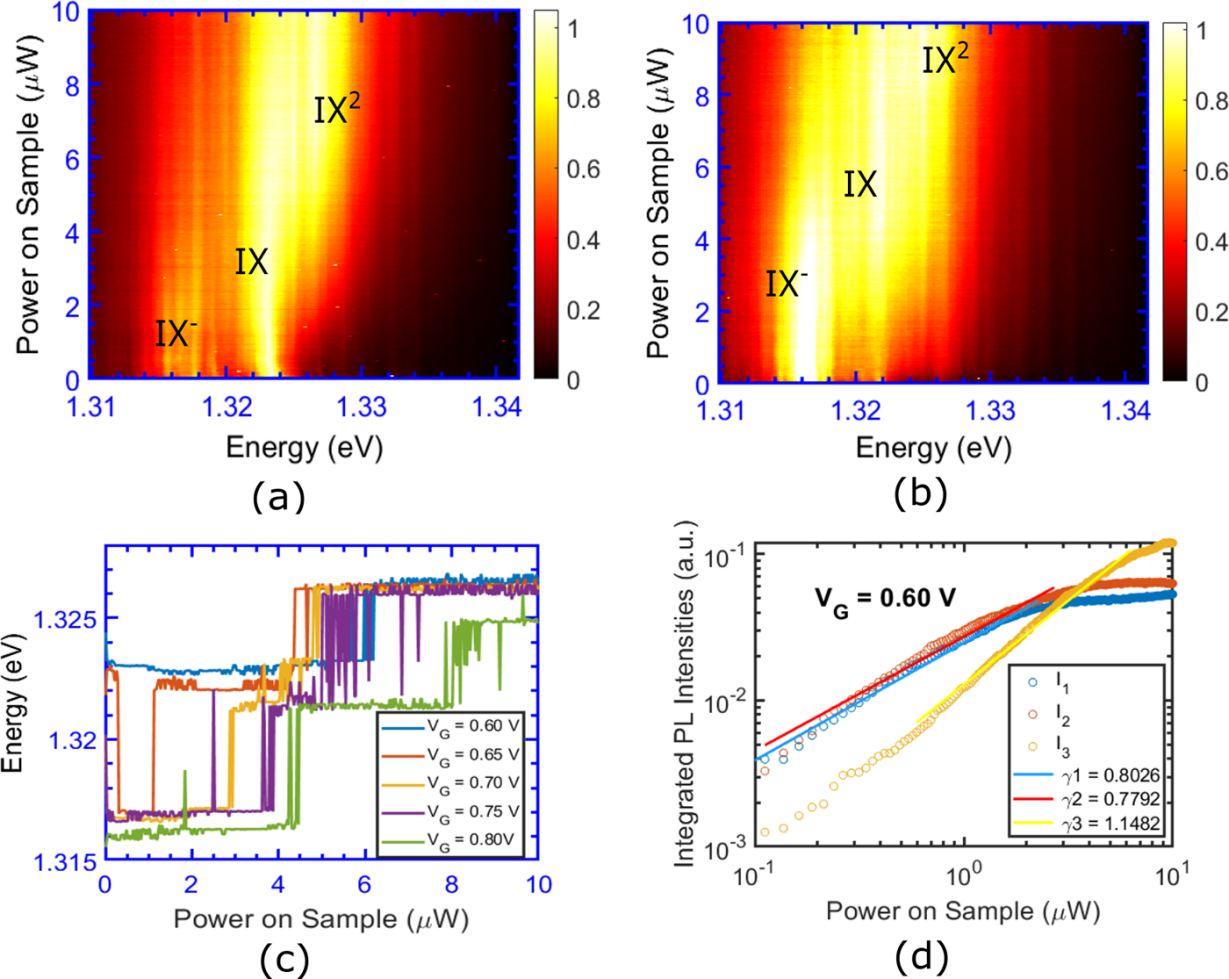
Normalized PL spectra vs power on sample at doping voltages of (a) *V*_G_ = 0.6 V, and (b) *V*_G_ = 0.8 V. (c) Traces of the peak PL intensity with excitation power at different doping voltages. (d) Power law fits of the integrated PL intensities of the three species, *I*_1_, *I*_2_, and *I*_3_ correspond to trions, excitons, and biexcitons respectively.

To confirm the nature of these bands, we fit the bands with separate Lorentzians with line widths of 2 meV each. The integrated PL intensities obtained from these fits are then plotted as a function of laser power and subjected to a linear fit in Figure 2(d). The highest energy band exhibits a growth coefficient of γ_3_ > 1. This superlinear growth confirms the biexcitonic nature of the highest energy band. On the other hand, the doping voltage dependence of the first band confirms that it has a trion origin. The polarity of the applied doping voltages confirms the negatively charged nature of the trions. We note that the small line widths of each emission band ∼ 2 meV is comparable to the smallest line widths reported so far, reflecting on the quality of the materials used and the sample quality. Our results show that at higher excitation densities, the PL emission from these samples is predominantly biexcitonic in origin. Moreover, the differences between the energies of the excitons and biexcitons, which arise from intratrap dipolar interactions, can explain most of the blue shift that is seen in the PL from these samples.

We next shift our attention to how the PL evolves with power as a function of the doping density. We trace the peak PL energies at different gate voltages in Figure 2(c). We are able to see the shifts in spectral weight from trions to excitons and biexcitons with increasing laser power. We also notice a DC stark effect, due to the nonzero electric field across the sample. The most exciting feature we observed was that the trion-to-exciton spectral weight jump was a function of the doping density of the sample and the trion emission dominated the PL spectrum up to higher carrier densities in the sample for larger doping densities.

In monolayers with itinerant or defect-localized excitons, at higher laser fluences, the exciton-trion ratio decreases.^35,36,38^ This is due to the prevalence of unbound free carriers. Trions can then form from excitons through electron capture^37^ from a sea of unbound charge carriers. This effect tends to increase with increased density of unbound free carriers at higher laser fluences, as the exciton-electron interaction increases.^43^ Localization, as seen in the case of excitonic quantum emitters based on defects,^36,43^ still allows this exciton-electron interaction. However, our studies reveal the absence of such a mechanism for Moiré localized excitons, suggesting a more robust localization, with the reduced center-of-mass motion^44^ resulting in a reduced interaction with the sea of free carriers. While the spatial extent of interlayer excitons may be larger, their effective interaction with surrounding carriers is reduced due to localization.

To understand the interplay between electrostatic doping and laser-created electron–hole carriers, we investigate how the exciton-trion transition for the case of a doping voltage sweep shifts with an increase in excitation intensity. In Figure 3(a),(b), we present the PL spectrum in a voltage sweep for two different excitation intensities. We see that the exciton-trion crossover occurs at a larger doping density at higher laser intensities. We plot the peak PL energy as a function of gate voltage for different excitation intensities in Figure 3(c); this allows us to see that the transition voltage shifts with increasing laser power. Figure 3(d) plots the transition voltages as a function of the laser power. We see that at higher laser fluences it takes a larger amount of dopant electrons to shift the dominant optical transition of the system to trions. The linear correlation between the doping voltage and the transition voltage suggests that any trions formed in the system are through the presence of doped electrons sitting at the Moiré traps.^45^

**Figure 3:**
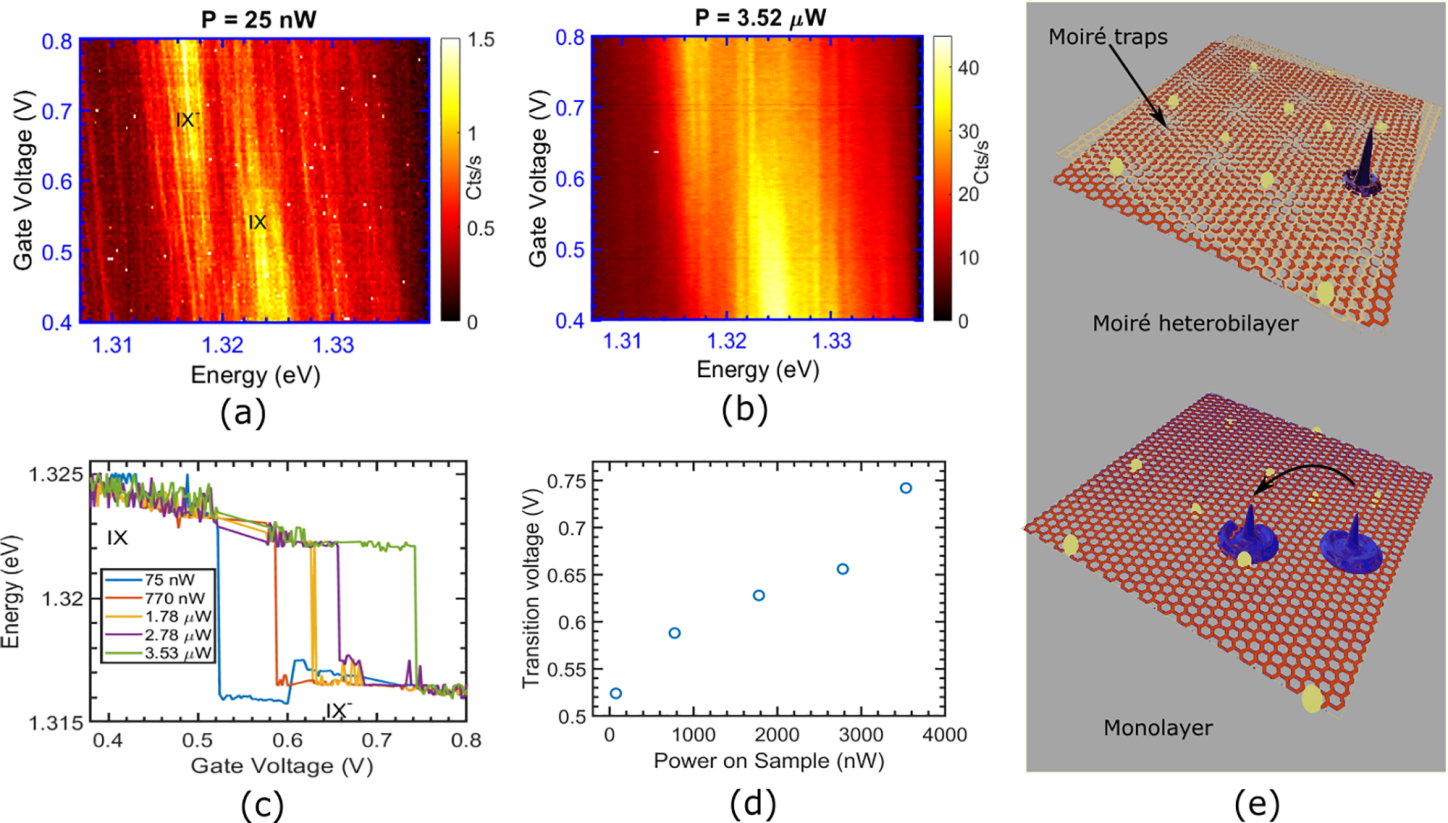
PL spectra with vs doping voltage at excitation powers of (a) 25 nW, and (b) 3.62 μW. (c) Traces of the peak PL intensity with doping voltage at different excitation powers, (d) transition voltages as a function of power on sample, (e) a cartoon illustrating the difference between highly localized excitons in a Moire trap and an itinerant exciton in a monolayer. Yellow spheres are free carriers and the blue hat profiles are representatives of the respective excitonic wave functions.

To summarize, our results paint the following picture. We start with an array of Moiré traps with a constant density of doped electrons at the trap sites. As we excite the system with a laser, the trion states, the lowest excited states, start filling up. This is when the PL is mostly trionic in origin. At higher laser fluences, as all the doped electrons are bound as trions, the absence of electron capture makes it impossible for the population of trions to grow; hence, the Moire traps start filling up with excitons. When all the traps are nearly filled, biexcitonic emission takes over. We note that the presence of charged biexcitonic species cannot be ruled out; however, further studies are required to mount evidence for their existence.

Our work illustrates the interplay of different species in the PL emission from MoSe_2_/WSe_2_ Moiré heterobilayers. Normalizing the PL spectra and tracing its evolution with excitation power show that most of the observed blueshift in these samples can be attributed to the spectral weight transfer in the PL across different species and hence to intratrap dipole–dipole interactions as opposed to interactions across different Moire sites. We find that at higher laser fluences (carrier densities < 10^12^ cm^–2^), the PL is dominated by biexcitonic emission. By analyzing the trion-exciton spectral weight, we also show that Moiré localization leads to an absence of electron capture in these systems, which hints at a qualitative difference in the strength of localization over conventional defect-based quantum emitters in monolayers. Investigating the time dynamics of trion formation and a rigorous rate equation analysis of these systems may shed more light on the exciton-electron interactions. Two-dimensional Fourier spectroscopy may also reveal how different trapped species are coupled to each other by analyzing the cross-couplings and shed more light on the excited state landscape in these systems.

## Supporting Information Available

Data confirming our results from a similar device as well as from an H-type hBL (PDF)
