# Supplementary material for: Interplay of Trapped Species and Absence of Electron Capture in Moiré Heterobilayers
Source: Nano Lett. 2023 Jun 23;23(13):5989–94. doi: 10.1021/acs.nanolett.3c01177 (PMC10347699; doi:10.1021/acs.nanolett.3c01177)
Supplement: Supplementary file 1 — nl3c01177_si_001.pdf [file nl3c01177_si_001.pdf]

Supporting Information for 'Interplay of trapped species and absence of electron capture in Moiré heterobilayers'

Arnab Barman Ray,† Arunabh Mukherjee,† Liangyu Qiu,† Renee Sailus,‡ Sefaattin Tongay,‡ and Anthony Nickolas Vamivakas*,†,¶

†*The Institute of Optics, University of Rochester, 480 Intercampus Dr, Rochester, NY 14627, USA*

‡*Arizona State University, 1151 S Forest Ave Tempe, AZ 85281, USA*

¶*Center for coherence and quantum optics, Department of Physics, University of Rochester, 480 Intercampus Dr, Rochester, NY 14627, USA*

E-mail: nick.vamivakas@rochester.edu

R-type samples, magnetic field measurements

We perform magnetic field experiments at low temperatures with our Attodry 1000 to confirm the R-type nature of our samples. Our experiment reveals the individual quantum emitters as they split and shift in energy with increasing magnetic field in Sfig. 1(a). In Sfig.1(b), fitting the PL maxima of an isolated emitter with a magnetic field, we arrive at a slope of about $0.0001989 \pm 1.4233 \times 10^{-5}$ eV/T. This corresponds to a g-factor of 6.87 $\pm$ 0.49, agreeing to what has been observed in Moiré heterobilayers with an R-type registry.[1,2]

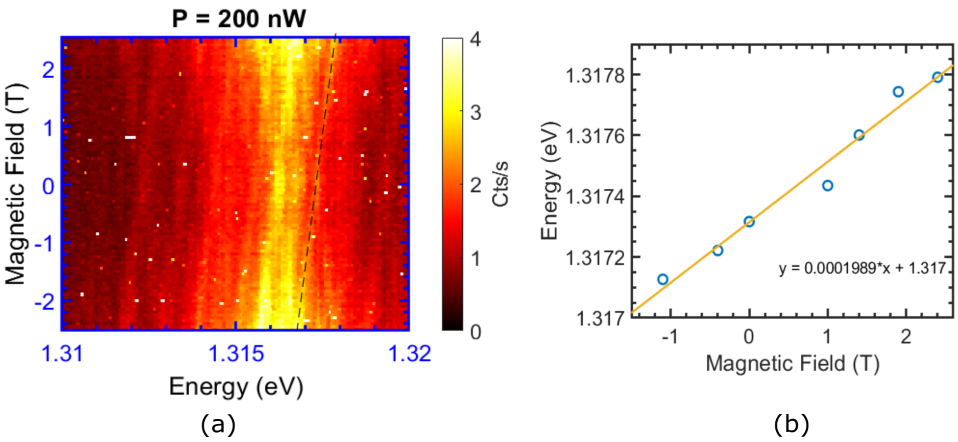

Supporting Figure 1: (a) Evolution of the PL spectra with magnetic field with low excitation power, emitter studied is traced with a dotted black line, (b) plot and linear fit of the energies of the emitter under question with magentic field.

Confirmation in a second sample

To confirm our results, we carry out similar measurements in a second R-type device. In this sample, too, we see a spectral weight transfer from trions to excitons as we increase the excitation intensity in Sfig. 2(a), suggesting the absence of any optical mechanism of trion generation in Moire heterobilayers in contrast to other kinds of systems. Secondly, Sfig 2(b) and (c) show the dependence of the PL on the doping density and reveal that the doping voltage required to affect the exciton-trion cross-over increases with increasing excitation density as more dopants are required.

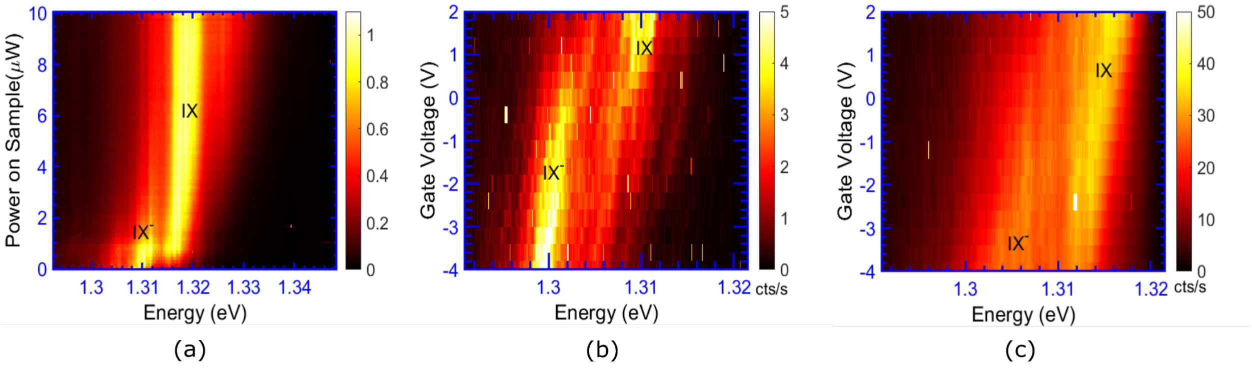

Supporting Figure 2: (a) Normalized Pl intensity with excitation power, (b) and (c) evolution of PL with doping voltage at 5 nW, and 500 nW respectively.

Emission in trapped species bands in an H-type sample

For a third H-type sample, we see the emission split into different bands corresponding to excitonic and bi-excitonic emission, respectively, similar to the case for an R-type sample. In H-type samples, the emission is dominated by both triplet and singlet excitons, while the emission from an R-type is mostly singlet in nature as the triplet is dark.[3] We see that with an increase in excitation intensity, there is a spectral weight transfer between the species in question. However, due to different degrees of inhomogeneous broadening, the emission's "band" nature is not as clear as the samples we have studied before. Sfig. 3(a) shows the normalized PL as a function of excitation intensity, while Sfig. 3(b) shows the spectral growth coefficient for each energy. The higher energy part of the spectrum is characterized by a superlinear growth rate confirming the presence of bi-excitons or even higher complexes. Note that the effects of power broadening and inhomogeneous broadening result in a smoother spectral growth coefficient than may be expected for separate energetic bands corresponding to different species.

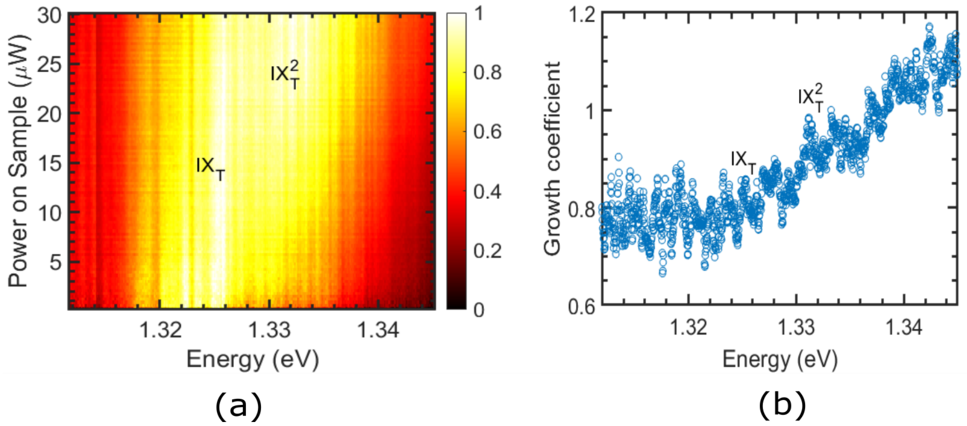

Supporting Figure 3: (a) Normalized PL intensity from triplet excitons in an H-type sample, (b) growth coefficient profile of the spectrum.

References

(1) Förg, M.; Baimuratov, A. S.; Kruchinin, S. Y.; Vovk, I. A.; Scherzer, J.; Förste, J.; Funk, V.; Watanabe, K.; Taniguchi, T.; Högele, A. Moiré excitons in MoSe2-WSe2 heterobilayers and heterotrilayers. *Nature Communications* **2021**, *12*, 1656.

(2) Holler, J.; Selig, M.; Kempf, M.; Zipfel, J.; Nagler, P.; Katzer, M.; Katsch, F.; Ballottin, M. V.; Mitioglu, A. A.; Chernikov, A.; Christianen, P. C. M.; Schüller, C.; Knorr, A.; Korn, T. Interlayer exciton valley polarization dynamics in large magnetic fields. *Phys. Rev. B* **2022**, *105*, 085303.

(3) Yu, H.; Liu, G.-B.; Yao, W. Brightened spin-triplet interlayer excitons and optical selection rules in van der Waals heterobilayers. *2D Materials* **2018**, *5*, 035021.
